# Finding Consensus on Trust in AI in Health Care: Recommendations From a Panel of International Experts

**DOI:** 10.2196/56306

**Published:** 2025-02-19

**Authors:** Georg Starke, Felix Gille, Alberto Termine, Yves Saint James Aquino, Ricardo Chavarriaga, Andrea Ferrario, Janna Hastings, Karin Jongsma, Philipp Kellmeyer, Bogdan Kulynych, Emily Postan, Elise Racine, Derya Sahin, Paulina Tomaszewska, Karina Vold, Jamie Webb, Alessandro Facchini, Marcello Ienca

**Affiliations:** 1 Institute for History and Ethics of Medicine Technical University of Munich Munich Germany; 2 College of Humanities École Polytechnique Fédérale de Lausanne Lausanne Switzerland; 3 Digital Society Initiative University of Zurich Zurich Switzerland; 4 Institute for Implementation Science in Health Care Faculty of Medicine University of Zurich Zurich Switzerland; 5 Dalle Molle Institute for Artificial Intelligence (IDSIA) The University of Applied Sciences and Arts of Southern Switzerland (SUPSI) Lugano Switzerland; 6 Australian Centre for Health Engagement, Evidence and Values University of Wollongong Wollongong Australia; 7 Centre for Artificial Intelligence Zurich University of Applied Sciences (ZHAW) Zurich Switzerland; 8 Institute of Biomedical Ethics and History of Medicine University of Zurich Zurich Switzerland; 9 School of Medicine University of St. Gallen St. Gallen Switzerland; 10 Bioethics & Health Humanities University Medical Center Utrecht Utrecht University Utrecht The Netherlands; 11 Data and Web Science Group School of Business Informatics and Mathematics University of Mannheim Mannheim Germany; 12 Department of Neurosurgery University of Freiburg - Medical Center Freiburg im Breisgau Germany; 13 Lausanne University Hospital (CHUV) Lausanne Switzerland; 14 Edinburgh Law School University of Edinburgh Edinburgh United Kingdom; 15 The Ethox Centre and Wellcome Centre for Ethics and Humanities Nuffield Department of Population Health University of Oxford Oxford United Kingdom; 16 The Institute for Ethics in AI Faculty of Philosophy University of Oxford Oxford United Kingdom; 17 Development Economics (DEC) World Bank Group Washington, DC United States; 18 Faculty of Mathematics and Information Science Warsaw University of Technology Warsaw Poland; 19 Institute for the History and Philosophy of Science and Technology University of Toronto Toronto, ON Canada; 20 Schwartz Reisman Institute for Technology and Society University of Toronto Toronto, ON Canada; 21 The Centre for Technomoral Futures University of Edinburgh Edinburgh United Kingdom

**Keywords:** expert consensus, trust, artificial intelligence, clinical decision support, assistive technologies, public health surveillance, framework analysis

## Abstract

**Background:**

The integration of artificial intelligence (AI) into health care has become a crucial element in the digital transformation of health systems worldwide. Despite the potential benefits across diverse medical domains, a significant barrier to the successful adoption of AI systems in health care applications remains the prevailing low user trust in these technologies. Crucially, this challenge is exacerbated by the lack of consensus among experts from different disciplines on the definition of trust in AI within the health care sector.

**Objective:**

We aimed to provide the first consensus-based analysis of trust in AI in health care based on an interdisciplinary panel of experts from different domains. Our findings can be used to address the problem of defining trust in AI in health care applications, fostering the discussion of concrete real-world health care scenarios in which humans interact with AI systems explicitly.

**Methods:**

We used a combination of framework analysis and a 3-step consensus process involving 18 international experts from the fields of computer science, medicine, philosophy of technology, ethics, and social sciences. Our process consisted of a synchronous phase during an expert workshop where we discussed the notion of trust in AI in health care applications, defined an initial framework of important elements of trust to guide our analysis, and agreed on 5 case studies. This was followed by a 2-step iterative, asynchronous process in which the authors further developed, discussed, and refined notions of trust with respect to these specific cases.

**Results:**

Our consensus process identified key contextual factors of trust, namely, an AI system’s environment, the actors involved, and framing factors, and analyzed causes and effects of trust in AI in health care. Our findings revealed that certain factors were applicable across all discussed cases yet also pointed to the need for a fine-grained, multidisciplinary analysis bridging human-centered and technology-centered approaches. While regulatory boundaries and technological design features are critical to successful AI implementation in health care, ultimately, communication and positive lived experiences with AI systems will be at the forefront of user trust. Our expert consensus allowed us to formulate concrete recommendations for future research on trust in AI in health care applications.

**Conclusions:**

This paper advocates for a more refined and nuanced conceptual understanding of trust in the context of AI in health care. By synthesizing insights into commonalities and differences among specific case studies, this paper establishes a foundational basis for future debates and discussions on trusting AI in health care.

## Introduction

### Background

The integration of artificial intelligence (AI) systems into health care is one of the most widely anticipated transformations of health systems worldwide [1]. AI promises improved diagnostics [2,3], optimized treatment strategies [4], and early identification of at-risk patients [5]. Prominent examples also include AI for assistive technologies offered to patients directly [6,7] and for informing public health decision-making beyond the individual [8]. More recently, large language model–based applications promise to revolutionize health care, with applications spanning from clinical research and processes to physician-patient relations [9]. Despite this range of potentially beneficial applications, the broader adoption of AI systems in health care has been struggling due to many inhibiting factors. Problems arise at the level of development, with data bottlenecks impeding the training of machine learning models or a lack of user-centered and value-sensitive design procedures affecting AI acceptability [10,11], and stretch to the level of practical implementation of AI systems [12-14]. Questionable improvements in real-life health care settings [15], a lack of regulatory frameworks [16], and unresolved questions of reimbursement [17] further complicate adoption of AI in health care.

In this paper, we focus on a central inhibitor of successful AI adoption in health care, namely, the low levels of user trust in AI systems [18]. Understanding and fostering trust in AI remains challenging, not only practically but also conceptually. Interpersonal trust constitutes a complex and contested construct in philosophy and social sciences [19-24]. Moreover, accounts diverge on what constitutes being worthy of trust, namely, on the definition of *trustworthiness* [19,20]. Unsurprisingly, there is also no consensus across disciplines such as computer science, philosophy of technology, and social sciences on the definition of trust in AI and the capabilities that a trustworthy AI should maintain [25,26]. As a recent publication assembling existing empirical literature on trust in AI and clinical decision support put it, “Different groups [of researchers], whilst seemingly agreeing in principle that ‘trust’ and ‘trustworthiness’ are important, can in fact be referring to very different concepts and talking past one another” [27].

The combination of fragmented conceptual research, practical concerns, and implementation difficulties inhibits fostering warranted trust in AI (ie, trust caused by the trustworthiness of the AI) [25,28], which will be increasingly crucial for delivering health care [29]. As recent empirical work using path analysis has highlighted, trust in AI-based technologies seems to have a significant effect on users’ intention to use such systems [30]. However, to build trust in—possibly trustworthy—AI systems in health care applications, we first need a conceptual understanding of trust in AI within the health care sector. This goal requires considering the diverse perspectives, expectations, and limitations that various research disciplines bring to the table in the context of human-AI interactions. Without conceptual clarity, attempts to foster the multifaceted concept of trust in AI—whatever it may *precisely* mean—run the risk of being inefficient or even detrimental, leaving users, including data scientists, physicians, and patients, vulnerable to placing trust in systems that may not warrant it or refraining from it in situations in which grounds for trusting actually exist, an occurrence of what we might call “unwarranted distrust” [31,32].

### Objectives

Bringing together international and multidisciplinary perspectives on the topic of trust in AI, this paper aims to provide common ground for defining trust in AI in health care as a reference point for future debates. To this end, we developed the first consensus statement on trust in AI in health care based on input from international experts on the topic drawing on iterative synchronous and asynchronous discussions of realistic case studies. Our results highlight the need for a more refined and nuanced understanding of trust in the context of AI in health care if the concept is to guide AI design and adoption processes and inform national and international governance.

## Methods

### Overview

Consensus statements from meetings of experts working on a particular topic constitute a common scientific approach across the fields of medicine [33,34] and ethics [35,36]. While they can take many different forms [37], their shared goal is to identify agreement among people working in a field and assemble multiple perspectives with peer-informed legitimacy [38]. The reporting of our findings follows the Accurate Consensus Reporting Document guidelines [37] and considered applicable elements of guidelines for reporting qualitative research [39,40].

### Recruitment

Recruitment for the consensus process took place as part of a 3-day workshop held at the École Polytechnique Fédérale de Lausanne in September 2022 in Lausanne, Switzerland. Authors GS, FG, AT, A Facchini, and MI, who organized the workshop, invited some participants directly based on their contribution to the field and to accommodate different types of expertise and backgrounds. They selected further participants from submissions to a call for abstracts for the workshop. Unanimous agreement concerning participants was achieved among conference organizers GS, FG, AT, A Facchini, and MI.

### Consensus Process

#### Overview

**Figure 1:**
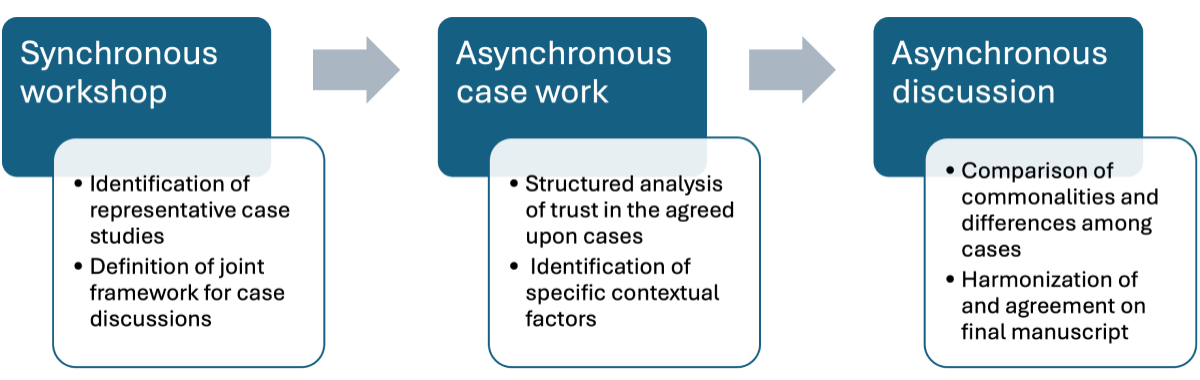
The 3-step consensus process and envisioned results.

The consensus process comprised 3 sequential steps involving both synchronous (step 1) and asynchronous (steps 2 and 3) activities. We describe them in the following sections. Figure 1 shows the steps of the consensus process and provides an overview of the intended outputs of each step.

#### Synchronous Phase: Selection of Case Studies and Framework

We conducted a workshop involving an international group of researchers from various disciplines. To arrive at a consensus regarding trust in AI in health care, we discussed the topic from complementary angles in a series of group activities that iteratively involved talks on specific aspects of trust, moderated group discussions, plenary sessions, and interactive panels. The moderated group discussions were spread over 3 days. They covered the topics of (1) conceptualizing trust in the context of human-AI interactions, (2) conceptualizing trust in AI in the context of health care specifically, (3) requirements for trustworthy AI in health care, and (4) recommendations for implementing requirements for trustworthy AI in health care. To achieve consensus on the case studies, all participants reviewed them. Only case studies assessed as representative yet distinct from other cases by all participants were promoted to the next step.

To guide our analysis, we made use of a conceptual framework. The framework was proposed by FG, critically discussed, and agreed upon by all authors. A conceptual framework represents a phenomenon in the form of a network with interlinked themes that describe how the phenomenon works [41]. Conceptual framework analysis provides an established qualitative method to guide the identification and organization of key aspects and relationships in contexts that draw on multidisciplinary bodies of knowledge [41]. We used framework analysis to provide structure and coherence of discussions for the asynchronous steps, systematically evaluating various dimensions of trust in AI in health care.

#### Asynchronous Step: Developing Case Studies on Trust in AI in Health Care

To understand how trust unfolds in the context of the agreed upon 5 cases, in the second step, smaller groups of authors expanded on the individual cases agreed upon in step 1. All contributors involved in the writing are listed as authors. Participants in this step were asked to explicate trust-relevant factors of the cases in accordance with the agreed upon conceptual framework. Responsibilities for coordinating the writing of individual case studies were distributed among the authors, with 1 person taking responsibility for leading each case study. KJ led the consensus for the first case study, JH led the consensus for the second case study, YSJA led the consensus for the third case study, GS led the consensus for the fourth case study, and JW led the consensus for the fifth case study. The results were compiled and shared by GS, FG, and MI.

#### Asynchronous Step: Comparison and Discussion of Case Studies

Having combined the individual case studies, we compared and discussed relevant aspects to synthesize our findings. We discussed our findings critically in light of existing literature and asked participants again to provide written feedback on the synthesized findings. This process was coordinated by GS, who wrote a first draft of the manuscript, which was then refined by FG, A Facchini, A Ferrario, and MI. All authors provided their feedback on the manuscript, which was incorporated and harmonized by GS and shared with all authors for approval.

### Ethical Considerations

In accordance with the existing regulatory framework in Switzerland, such as the Human Research Act and the local rules at the École Polytechnique Fédérale de Lausanne as hosting institution, no ethics approval was required for this collaborative research project involving only consenting experts in the field.

## Results

### Overview

The results of our study unveil the complexities of understanding trust in AI within the health care domain. Our findings represent consensus among international experts guided by conceptual framework analysis. The following sections provide the characteristics of the involved experts and report the findings from the individual synchronous and asynchronous steps of our consensus process.

### Recruitment

The characteristics of the 18 participants in the consensus process are described in Table 1.

**Table 1:** Participant characteristics—multiple entries possible for location and background (N=18).

Characteristics			Participants, n (%)
**Gender**			
	Women	7 (39)	
	Men	11 (61)	
	Nonbinary	0 (0)	
	Prefer not to say	0 (0)	
**Geographic location**			
	Europe	16 (89)	
	North America	2 (11)	
	Oceania	1 (6)	
**Background**			
	Computer science	6 (33)	
	Ethics	5 (28)	
	Medicine	4 (22)	
	Philosophy	8 (44)	
	Public health	2 (11)	
	Social science	3 (17)	

### Consensus on Trust and Trustworthiness

#### Synchronous Phase: Selection of Case Studies and Framework

A total of 5 case studies emerged from the synchronous group discussion and were identified as paradigmatic examples of the various applications of AI in health care, namely, diagnosis, clinical risk assessment, public health surveillance, assistive technologies, and health care resource allocation. The involved experts agreed that these cases may not provide an exhaustive sample of all potential AI applications in health care yet allow for a meaningful representation and comparison of trust in AI across largely different health care settings.

To guide the asynchronous analysis of the individual cases, all involved experts identified and defined key components of a conceptual framework for trust in AI in health care. We developed this framework with the backdrop of existing conceptual work on trust in data use in health care, preliminary discussions of the shortcomings of present conceptual work describing what trust in AI is, and existing guidance on conceptual framework development for research and scale development in medicine [29,31,42-49].

Following existing conceptualizations of trust in the context of data use in health care [47], themes were grouped into two main areas: (1) the context of trust and (2) causes and effects of trusting. We considered trust as a context-specific, relational construct between 2 actors that is shaped by the environment in which it develops over time [50]. Therefore, our framework implemented the context specificity of trust by considering (1) the environment within which an AI system is deployed (ie, the health care application in which the AI operates), (2) the actors involved in the specific trust relationship, and (3) the factors that frame the trusting relationship. Here, framing factors influence “the process by which people develop a particular conceptualization of an issue or reorient their thinking about an issue” [51]. Examples of framing factors are historical context, cultural aspects, norms and values, fears, public sentiment, overarching belief systems, and religious beliefs. In addition to context, our framework includes factors that support or inhibit trust as well as the effects of trusting AI systems. Causally important themes for trusting relationships typically relate to factors that make AI trustworthy or untrustworthy in the eyes of those placing trust. Examples are the reliability and accuracy of the AI and, arguably, the level of interpretability of its outcomes [28,52]. In summary, our framework comprised 5 themes: environment, actors, frames, causes, and effects (Textbox 1).

**Conceptual framework for warranted trust in artificial intelligence (AI) in health care.**

**Context of trust in AI in health care**

- Environment: What is the setting for which the AI system is intended?
- Actors: Who are the involved actors in the trust relationship?
- Frame: What frames trust in relation to AI?

**Cause and effect of trust in AI in health care**

- Cause: What makes AI trustworthy?
- Effect: What is the effect of trust in relation to AI?

#### Asynchronous Step: Developing Case Studies on Trust in AI in Health Care

Drawing on our framework and previous research, smaller groups of authors examined particular aspects of AI used for diagnostic purposes, clinical risk assessment, public health surveillance, assistive technologies, and health care resource allocation. The results of the individual groups led by KJ, JH, YSJA, GS, and JW, respectively, are reported in the following sections.

##### Diagnostic AI in the Clinic

Machine learning and, more specifically, deep learning have proven to be particularly suitable for computer vision, especially image processing and pattern recognition. Important preconditions to apply these techniques in medicine, such as suitable infrastructure or availability and storage of digital images, are also being increasingly met, at least in high-income countries. This may explain why most current and proposed AI systems in medicine are used to aid image-based diagnostics in fields such as radiology, ophthalmology, and pathology [53-55]. As a large part of the workload of, for instance, radiologists is to interpret medical images [56], diagnostic AI systems could increase this capacity; support or take over the tasks; and, thereby, change the organization of image-driven diagnostics [57].

Some studies have indicated that, under specific circumstances, AI systems achieve accuracy that is at least equal to that of expert radiologists and pathologists or even outperform them when detecting, classifying, and segmenting tumors in ultrasonography, x-ray imaging, magnetic resonance imaging scans, and digitized microscopy slides [58,59]. These findings have raised massive enthusiasm and fueled the motivation to develop and use algorithms in image-driven diagnostics. As a particular example, we will focus on a recently approved AI system for the diagnostic analysis of chest x-rays [60].

The system aids diagnosis by analyzing chest x-rays, the most frequent radiological examination worldwide. It does so by classifying the scans in nonpathological and potentially pathological cases. For the first group, the program provides a fully automated report, removing the necessity for any further follow-up on the image by radiologists. The scans of the second group are forwarded to trained physicians for further radiological analysis. A test of the software on approximately 10,000 chest x-rays from Finland showed a very high sensitivity (99.8%) with a low specificity (36.4%), yielding a very low probability of missing any critical findings [61].

However, the use of diagnostic AI is associated with technological obstacles as well as epistemic and ethical challenges [62]. Although the potential of self-supervised learning to generate expertlike annotations has been demonstrated, an insufficient number of expertly annotated diverse images is currently often a limiting factor in training AI models on a technical level [63]. Large datasets, for instance, in pathology, can also pose problems if downsampling leads to crucial information being lost, demanding different kinds of representation [64]. Beyond such technical difficulties, there are also more fundamental obstacles, including the fact that diagnostic tasks are not usually choices between 2 distinct outcomes [65]. Another concern is the frequent lack of explainability of medical AI, which limits physicians’ ability to recheck the AI’s output [66]. It has been argued that this raises particular challenges as decisions based on black-box AI systems are potentially not fully interpretable for the human physician. Moreover, when offering explanations, it is essential to consider the epistemic requirements of users (eg, radiologists and patients). Explanations that lack a clear connection to clinical knowledge despite being technically accurate fall short in delivering the desired support for clinical practice. Similarly, patients who wish to understand the basis of their diagnosis may find explanations that diverge from their own illness experiences unsatisfactorily [67,68]. That said, there is empirical evidence that a lack of explainability is not necessarily perceived as a problem by physicians if a diagnostic system has been properly validated and other detailed information about the AI system is available [69,70].

The case discussed here takes place in a clinical environment of image-driven diagnostics. It directly involves medical professionals and the AI system, whereas patients do not interact with the system itself; in addition, the developing company and regulatory bodies are indirectly involved. Framing factors are discourses about job security in radiology and the danger of physicians being replaced by AI [71]. Trust leads to acceptance of the system by physicians, potentially at the cost of deskilling, and is arguably fostered by the accuracy of the diagnostic program in question [72].

##### Predictive AI for Clinical Risk Assessment

AI algorithms can be used to predict individual patient trajectories. Examples include the prediction of COVID-19 severity [73], the prediction of delirium [74], prognostic models of respiratory diseases [75], or systems predicting future lung cancer risk [76]. In addition, predictive models are crucial for determining individualized treatment plans, often particularly relevant in oncology [77]. The difference from the diagnostic use case is that the prognostic case concerns the future (ie, informs decision-making under situations characterized by an inherently larger uncertainty). The prognostic prediction informs choices of action, treatments offered, support, and resource allocation and, therefore, can, in turn, influence the very outcome that has been predicted [78].

As a specific case study, let us consider the prediction of circulatory failure in intensive care units (ICUs) [5]. In ICUs, a great number of machines monitor the state of patients and need to be observed constantly as patient conditions may worsen rapidly. The existing technologies for life support and vital sign monitoring provide information and alerts that clinicians need to oversee. However, the high rates of alerts, including false positives, may cause alert fatigue for practicing clinicians, impairing optimal care. In this context, systems that predict severe deterioration more accurately can obviate alert fatigue and potentially lead to better patient outcomes. The system reported in the study by Hyland et al [5] integrates information from multiple organ function–monitoring systems to alert clinicians to potential circulatory failure 8 hours in advance. Note that the information available to the system is necessarily incomplete, and it is impossible to predict future circulatory failure with 100% accuracy using any algorithm. However, the system in question successfully predicted 90% of cases of circulatory failure in the test set and 82% of them >2 hours before the event [5], so it would likely prove useful in a clinical setting.

The system described here can only be deployed in the environment of hospitals or clinics. Physicians, nurses, and potentially caregivers may interact directly with the AI system, and the social circle of a patient could also potentially receive information derived by the AI system, whereas the patients admitted to an ICU will likely not interact with the system itself. Critical contextual factors are the system’s use in stressful situations with the risk of severe consequences, sometimes under time pressure, and the need to synthesize a multitude of complex information. Trust in such a situation can be built by overall improved outcomes in settings where the algorithm is used, ideally by investigating it through a randomized controlled trial. In addition, there should be reasons to believe that the AI system is not biased or will not lead to harmful consequences for any subgroups of the population and that it produces reliable individual decisions that are robust to design choices [79,80] or randomness [79,81] in the AI pipelines. Trust built on these premises will promote AI’s acceptance and use. However, as with other medical interventions aiming to change the future, such as screening programs [82,83], trust could also lead to worse outcomes if the system does not actually deserve it (ie, if it is not *trustworthy*).

##### AI for Public Health Surveillance

Public health surveillance involves the identification of signs of population-level health anomalies and potential disease outbreaks from a heterogeneous collection of data sources [8]. With its intrinsically data-driven nature, public health surveillance has increasingly become a domain for AI applications as AI provides a range of novel methods for data collection and data analysis in large and varied samples [8]. A comprehensive survey of the literature showed that the COVID-19 pandemic highlighted the potential of AI systems in improving surveillance of infectious disease outbreaks [84].

Consider the example of EPIWATCH among the numerous AI-based surveillance systems that provide early signs of disease outbreaks [85]. Developed at the University of New South Wales in Australia, EPIWATCH is an open access web-based tool that offers an interactive dashboard with a sortable, searchable, and filterable global map based on 30 days of data [85]. The tool has been successfully used to model and identify patterns of disease outbreaks, providing crucial information on risk factors and geographic distribution.

For instance, using publicly available data, EPIWATCH has been used to trace global Zika virus outbreaks, tracing transmission modes, affected countries, and complications such as microcephaly [86]. Similarly, a different group of authors used EPIWATCH to model the global epidemiology of hepatitis A, identifying the United States and Europe as major centers of outbreaks, and provided quantitative global data on the most common risk factors, which seem to be homelessness and foodborne outbreaks [87]. Such data can be helpful to inform policy making at both a global and local level.

Let us summarize the contextual factors of this case. Contrary to the previous examples, AI systems for public health are deployed in nonclinical settings. This web-based example is potentially accessible by anyone with a computer or mobile phone with an internet connection interested in data analysis to predict disease outbreaks. Therefore, relevant actors include the developers as well as a broad spectrum of potential users, ranging from public health practitioners and policy makers to the public. Important framing factors for trust are the severity of the modeled disease as well as the stage of the epidemic or pandemic, impacting the relevance and acceptability of the tool. As the COVID-19 pandemic has demonstrated, antiscience sentiments and conspiracy theories contribute to how public health tools are viewed. Finally, usability aspects further influence whether information will be taken up by practitioners. Trust in the application will likely lead to increased uptake among decision makers. The trustworthiness of the platform developers and endorsement by authorities, such as public health experts and policy makers, will further determine whether the wider public will trust the model’s output.

##### AI for Assistive Neurotechnology

In comparison to the examples discussed previously, a quite different trust relationship can be found in the case of assistive neurotechnologies based on brain-computer interfaces (BCIs). BCIs can, for instance, be used for neurorehabilitation, restoring lost function, or augmenting and enhancing existing cognitive or motor abilities [7]. AI, especially data-driven machine learning with deep neural networks, plays a crucial role in their development and use as these methods enable and facilitate the modeling and decoding of complex neural signals as well as the adaptation to individual users [88]. To delineate differences in the trust relationship between AI-based neurotechnology and other medical AI, we focus on a specific example: a BCI-guided robotic hand orthosis for rehabilitative purposes.

Orthoses are external wearable devices that can be “used to compensate for impairments of the structure and function of the neuromuscular and skeletal systems” [89]. In line with numerous existing research projects at the level of preclinical prototypes [90], let us consider a specific BCI-based robotic orthosis that allows patients with stroke to restore some volitional control of hand-grasping movements. To do so, the system records electrical activity of the dominant motor cortex noninvasively through an electroencephalography cap and extracts features from the measured signals using machine learning–based classifiers to extract patient-specific neural markers of intended hand movements, which are then translated into mechanical movements of the robotic hand orthosis.

There are several aspects of such personalized AI-based assistive neurotechnologies for neurorehabilitation that set them apart from medical AI used for diagnosis, prediction, or public health surveillance. One crucial difference that stands out is that the AI in question is *embodied* in the sense that the AI used for decoding neural signals and for translating them to mechanical movement is inextricably linked to a physical object—namely, the hand orthosis. This implies that physical design aspects play a crucial role on whether trust is expedited to the device [91].

However, there are further differences that impact the trust relationship involving actors, the environment, and framing. First, assistive rehabilitative neurotechnology is designed for patients with impairment of cognitive or motor functions who need to engage with the technology. These patients are also the main trusters of this technology, not health care professionals or public health experts. Second, suppose the neurotechnology also helps patients in their everyday life. In that case, it should not only work under highly constrained and controlled laboratory or clinical conditions but also in a patient’s home. This implies additional demands for the devices’ ease of use and their security in a more open environment, which would be crucial for the trust of bystanders in these devices. Third, due to the BCIs’ physicality, trust in assistive neurotechnology may also be framed differently, invoking widely known images from science fiction [92]. Fourth, users may be concerned that decoding their brain activity could be used not only to control the robotic orthosis but also to infer other types of information that would not be available using other sensing technologies, thus raising potential threats to their privacy. Therefore, addressing potential fears of patients and designing AI that assures human control (in the sense of “human in the loop”) at any moment adds to the complexities of building trust in such embodied AI.

To summarize, the assistive AI system discussed here is embedded in an environment of clinical neurorehabilitation. Its trusters are primarily patients and rehabilitation experts who trust in the developing engineers and the pertinent regulatory bodies. In the context of embodied AI, many specific framing factors influence its perception, especially from science fiction literature and cinema. It is also shaped by public attitudes and policies on related technologies such as robotics. To build trust in this environment and prove the accuracy of the AI, a lack of conflicts of interest on the part of the developers; sufficient understanding of the underlying technology by its users and rehabilitation experts; and independent, long-term technical support seem key. Successful trust building will result in wider acceptance and adherence by users, as well as potential inclusion of the technology in public health programs, facilitated regulatory compliance, and improved insurance reimbursement. Taken together, these elements can facilitate access and increase the technology’s affordability by expanding the number of its users.

##### AI for Health Care Resource Allocation

A 2019 study in *Science* revealed that software provided by the health service company Optum, and which was being used to manage the care of >200 million patients at hospital centers across the United States, was significantly—even if unintentionally—biased against Black patients [93]. The machine learning algorithm aimed to predict the future health care needs of patients and direct extra medical care toward the most vulnerable. However, it was shown to systematically underestimate the needs of Black patients. It did this because it used health care costs as a proxy for need in its risk score despite health care costs not being a neutral and reliable proxy for health needs in this context. This led it to assign consistently lower risk scores to Black patients compared to White patients who were equivalently sick. This was despite the model being “race blind” in the sense that race was not specified in the input data [94].

When considering how this case study relates to trust in and trustworthiness of AI in health care, it is important to note that this is not a case of AI being introduced into a dyadic physician-patient relationship but, rather, of its integration into the operations of complex health systems involving many actors and institutions. Therefore, philosophical accounts of trust that focus on the properties of individuals—these accounts traditionally comprise reliability *plus* some appropriate motivational state on the part of the trustee toward the truster, for example, goodwill [95]—are insufficient to capture the conditions necessary for trustworthy AI in these contexts.

Accounts of institutional trustworthiness are more useful here. They often emphasize features such as transparency as well as competence and reliability [96]. Accounts of trustworthiness that do not necessarily require a phenomenological state such as goodwill may also be useful. For example, the trust responsiveness account by McGeer and Pettit [97] emphasizes the importance of an agent—or, in our case, institutions—responding appropriately to the reasons for doing what they are being relied upon to do. Alternatively, one might provide a deflationary account that either equates trustworthiness with reliability [48] or rejects trust as an appropriate attitude altogether [98].

What does it mean to meet these conditions in this and similar cases of integrating machine learning into resource allocation decisions across an entire hospital or health system? Trust responsiveness demands that systems such as the one provided by Optum be introduced to promote health and well-being and improve patient outcomes and not to meet other goals such as cutting costs to maximize profits for insurance companies and health systems. Using a system such as Optum may not necessarily require internal algorithmic transparency, which is often the focus of work considering the ethics of machine learning algorithms in health care [99], yet may be difficult to translate into a genuine understanding of the AI system by clinicians or patients. However, there needs to be openness regarding design decisions and value choices involved in the algorithm’s construction and implementation [100]. Demonstrating these may require engaging in ethical and algorithmic impact assessments during the design process [101,102], welcoming algorithmic auditing after implementation [49], and abiding by regulatory frameworks throughout the product life cycle [103]. Ideally, transparency for AI systems for resource allocation would highlight both global explanations [104], given that the goal of the algorithm is to distribute resources across a population [105], and local explanations for individual decisions as the biased program impacts individual care. Both are potentially useful for promoting fairness [104].

In addition, it is crucial to consider contextual factors impacting such trust relationships. The program was developed by a for-profit health service provider in the US health care system and used to determine resource allocation. Key actors were the health service company itself, which provided the algorithm; the health systems; clinicians interacting with the algorithm’s recommendations; patients having their care influenced by the algorithm’s recommendations; and, finally, regulatory bodies and (internal and external) algorithmic auditors. Important framing factors include media reporting on algorithms and their impact and, on a conceptual level, theories of institutional trust. Such trust, though unwarranted here, would result in the model’s acceptance and use. It is supported by an AI’s reliability and competence as well as by the transparency of the developers concerning design choices and the values reflected in them.

#### Asynchronous Step: Comparison and Discussion of Case Studies

As the 5 cases highlight, medical AI is a multifaceted concept encompassing a diverse group of systems, environments, actors, and framing factors. Talking about trust in medical AI in general can only be a first approximation to the phenomenon. However, some general elements of trust were accepted by all experts. First, they agreed that trust provides a way to deal with complex situations in the face of uncertainty [106,107] and is established in anticipation of a beneficial outcome. Therefore, in a rather minimal fashion, and in line with existing literature, an agreed upon definition of trust in AI in health care focuses on giving discretionary power to an AI system with respect to a specific health care–related task [29,108]. At the same time, it was agreed that situations of trust were characterized by risks, rendering the trusting agent vulnerable to betrayal of trust [21]. To minimize such risks, potentially of life and death in health care, experts agreed that we need to only promote warranted trust in medical AI (ie, trust that is justified, plausible, and well grounded [19,28,49]). In the literature as well as in regulatory contexts, AI systems that warrant such trust have been described as trustworthy [49,109,110]. Therefore, in the eyes of the experts, a complete assessment of trust in AI includes epistemological and practical considerations of trust as much as examining the trustworthiness of the AI in question (ie, the functionalities of an AI system that morally and practically vindicate a relationship of trust [111]).

The involved experts agreed on many practical shortfalls of current AI in health care potentially impacting its trustworthiness. These include the lack of explainability of many AI algorithms [28,66,112]; the difficulties of benchmarking model performance [113]; or the longtail of exceptional situations that will be difficult to anticipate at the development stage, from nonstandard data inputs to the system’s deployment in a health care system for which it was not originally trained [114]. In the eyes of the experts, anticipatory difficulty also relates to risks of AI perpetuating, hiding, or reinforcing statistical and social biases as well as other data problems [115,116]. Finally, it was stressed that many medical AI tools so far lack proper engagement with stakeholders in their development, which is crucial for ensuring that they adequately address a real clinical problem and identifying their relevant ethical implications [117].

When looking at the individual case studies, commonalities and differences among them arose at micro and macro levels. Table 2 provides an overview of the contextual factors of each case study.

**Table 2:** Contextual factors for the 5 case studies.

	Environment	Actors	Framing factors	Causes of trust	Effects of trust
Diagnostic AI^a^ (chest x-rays)	Image-driven diagnostics (radiology)	Medical professionals and AI system; patients to a limited extent	Discourse regarding job security and potential AI replacement	Accuracy, design transparency, and human competencies and virtues	Acceptance of systems by physicians, potentially at the cost of deskilling
Predictive AI (ICU^b^ setting)	Clinical setting of an ICU	Physicians, nurses, and AI system; patients to a limited extent; and potentially caregivers and family members	Stressful situations potentially with a need to act under time pressure, risk of severe consequences, the need to synthesize too much information, and alert fatigue	Accuracy, transparency, and explainability; fairness; exclusion of harm; and rigorous testing (eg, in the form of an RCT^c^)	Acceptance and use of the system, potentially at the risk of erroneous clinical decisions following misleading predictions
Public health AI (disease outbreak model)	Nonclinical setting—publicly accessible web-based tool for the analysis of heterogeneous data	Developers, public health practitioners, policy makers, and the public	Stage and severity of disease outbreak; usability aspects (eg, intuitive interface or data visualization); and, potentially, antiscience sentiments and conspiracy theories with regard to the disease and health service providers	Historical accuracy and endorsement by authorities	Acceptance and use of the system by public decision makers (public health experts and policy makers)
Assistive AI (neurorehabilitation)	Clinical neurorehabilitation; elective use of different technologies for different activities, potentially every day	Patients and their caregivers and social circle, potentially including employers, engineers, and regulators	Clinical setting, science fiction literature and cinema, and public attitudes and policies on related technologies	Accuracy, privacy, lack of conflicts of interest, independence, long-term technical support, and user understanding of the underlying technology	Technology acceptance by users, health care professionals, and health care providers; potentially facilitated reimbursement and increased affordability and accessibility
Resource-allocating AI (predicting costs and needs)	Health service providers and health care system	The developing company providing the algorithm, the health system implementing it, the clinicians interacting with it, the patients having their care influenced by the algorithm, and regulatory bodies and algorithmic auditors	Media reporting on algorithms and their impact and theories of institutional trust	Reliability, accuracy, transparency, design, and model-centric explanations	Acceptance and use in health care systems

^a^AI: artificial intelligence.

^b^ICU: intensive care unit.

^c^RCT: randomized controlled trial.

Most commonalities among the different cases can be found with regard to their causes and effects, some of which arise from the very structure of trust. A common effect of trust was found in it leading to wider acceptance of the technology in question. Such acceptance implies an increased uptake by its intended end users, resulting in a larger role in health care systems, facilitating access, and potentially increasing the systems’ affordability. In line with the literature, common aspects with regard to trust-supporting features included both aspects intrinsic to the system, such as transparency and explicability, and extrinsic factors, such as proper external validation and assessment of potential biases [25,29].

Across the discussed cases, some forms of transparency were identified as supportive of establishing trust. Such transparency included openness by the developers about their measures of a well-working system, enabling an alignment with the goals and values of patients and clinicians. Promoting transparency was also assessed as a good way of promoting trust among clinicians using the systems. For instance, there is some empirical evidence that being transparent about the functioning of a triage system makes clinicians more likely to trust it [118]. The extent to which this is possible depends on the exact nature of the system, with clear differences among algorithms that are interpretable by design [119], algorithms that can be explained in terms of their exact functioning on the level of predictors and their weights, and algorithms whose decision process cannot be interpreted causally.

Informational openness was also recognized as central to promoting a system’s external trustworthiness (eg, if a system’s reliability and performance metrics are publicly available). From the discussed cases, it also emerged that rendering information accessible to all relevant auditing bodies is crucial for mechanisms of accountability and, in doing so, for creating the necessary epistemic basis for trust at an individual level as much as among the public, who can have confidence that the AI systems have been subject to meaningful scrutiny. In this sense, transparency was deemed to support trust among affected communities and stakeholders by providing the means to evaluate claims made about these systems with regard to bias and discrimination. AI systems reproducing inequalities and exacerbating injustices may, in turn, erode public trust not only in the AI-based tools themselves but also in stakeholders involved in building, deploying, and using such systems. Addressing and testing for such biases was deemed particularly relevant considering that “personal and collective experiences with discrimination or degradation-along lines of race, class, gender, or other personal characteristics especially create reasons for suspicion if not outright distrust” [99]. However, as the experts agreed, such testing needs to go beyond the sole measure of excluding apparently discriminatory information. Deep learning models may predict a patient’s race from medical images such as chest and hand x-rays and mammograms [100] or identify patient self-reported race from redacted clinical notes [101] despite human experts being unable to do the same. Therefore, as indicated by the resource allocation case study, discrimination can occur even when models are apparently *race blind* through implicit proxy features, as is well understood and documented in algorithmic fairness research [120]. Moreover, discrimination through differential impact across subpopulations might occur precisely because models are insufficiently sensitive to inequitable distribution of social determinants of health along ethnic or racial lines [121].

Beyond these common themes, our cases highlight important contextual differences among trust in different kinds of medical AI. In particular, these relate to (1) framing factors, (2) previous knowledge of the targeted trusters demanding different levels of explicability and transparency, and (3) the different risk-benefit trade-offs in different environments. Sometimes, the risks in medicine are grave for individual patients, such as in an ICU setting. However, in the resource allocation setting, the risk-benefit trade-offs concern both individual-level consequences of allocating or denying a resource to an individual and group- or population-wide benefits or harms due to better or worse resource use.

## Discussion

### Principal Findings

Using conceptual framework analysis, our study provides the first consensus-based publication investigating trust in AI in the domain of health care. Commonalities and differences emerged from the examination of our case studies, particularly with respect to causes and effects of user trust. Across all cases, trust was found to enhance acceptance and adoption of AI technologies, and some factors such as a system’s accuracy were similarly deemed to be crucial for the trustworthiness of all discussed systems. Developers’ openness about a system’s internal workings, assumptions, and value judgments underlining the technical choices were also considered crucial across the case studies. Such transparency fosters alignment with the goals and values of health care professionals and patients; supports accountability mechanisms; and may help address biases and discrimination, which is essential for building trust among affected communities and stakeholders. However, our analysis also highlights contextual differences among trust in various forms of AI in health care. These differences relate to a multitude of case-specific aspects, such as framing factors, previous knowledge of the trusters, and risk-benefit trade-offs in different environments, necessitating tailored approaches to establish trust and trustworthiness. This finding has implications for anyone aiming to foster trust in AI within the health care domain, from developers and health care professionals using AI systems to public health experts and regulators—trust-building measures should distinguish among different types of AI systems considering not only implied risk levels and legal responsibilities in case of errors but also the factors outlined in our conceptual framework: context, actors, discourse, and the mechanisms of trust building.

### Implications

Our findings have implications for developers, patients and health care professionals, and regulators aiming to increase trust in medical AI systems. The developing companies should communicate openly about the algorithmic design and training of their AI systems and tailor their level of transparency to the communicative needs of their respective audiences [122]. Such needs will be very different depending on whether the AI is used by patients directly (assistive AI case), trained physicians (diagnostic and predictive AI cases), health economists and policy makers (resource allocation AI case), or potentially the interested public (public health AI case). Individual AI end users need to be educated on the technology’s limitations and carefully consider who bears the risk and responsibility of involving the AI—especially if it is not themselves.

At a regulatory level, distinguishing among different kinds of AI systems in medicine seems also crucial, regulating them according to the level of implied risk as currently proposed by the European Union AI Act—or possibly at a finer-grained level specific for health care [123]. Regulation is also needed to clarify who bears legal responsibility in the case of specific errors. While there is a clear responsibility by developers to avoid systematic errors as much as possible, for the foreseeable future, it will not be possible to design an AI system without any errors. Therefore, some responsibility also falls on health care providers who use the systems as part of their workflows to use them appropriately and with the right level of human oversight. This requires strengthening both the technical capabilities of health care practitioners to evaluate AI systems and the responsibilities placed on the developers of systems to openly document where a system may be expected to generate errors. Given the limits of both, proper certification of AI systems is furthermore crucial, allowing for trust in an overseeing institution to be extended to the system itself [124].

A final important point is that there should be a focus on institutions implementing these tools in a trustworthy manner, not on increasing trust. There are several reasons for this. First, trust could be increased through marketing and presentational gimmicks targeted at patients and clinicians, which would do nothing to increase trustworthiness [105]. Second, trust may be hard to achieve given prevailing and justified distrust in health service providers such as Optum and private health systems and marginalized communities’ distrust in health care systems due to historic or entrenched disparities in outcomes or experiences of care [125]. In contrast, this may be less of a problem where there are higher levels of trust in health institutions that might implement AI systems [106]. Finally, the size and complexity of health systems complicate questions of where, why, and in whom the individual patient should place trust within these systems [87]. This creates challenges in assessing how far the design and implementation of an algorithmic system for resource allocation impacted the attitudes of trust in affected patients.

### Limitations

While our findings aim to provide a starting point for fruitful further work on AI in health care, there are several limitations to our consensus process and its results. Of these limitations, 3 are related to the selection of experts for our consensus process. First, owing to the recruitment of participants working on trust in AI in health care, our work is biased toward a position that considers trust a useful and meaningful concept in the context of AI in the first place. While this position is supported by a substantive part of the literature [28,31,49,52], it should be noted that there are also outspoken critics of using the notion of trust with respect to AI in the first place [98,126,127]. Second, given the location of our workshop, our recruitment focused largely on experts from Europe, with only a small addition of participants from North America and Oceania. While this may reflect the central role that trust and trustworthiness have taken in regulatory debates across the European Union, it limits the generalizability of our findings to other contexts. A recent systematic review of empirical research on trust in AI also revealed a lack of diversity in the discussion surrounding this topic [26]. This review underscored the need for a broader range of perspectives to more comprehensively understand and address the complexities of trust in AI systems [26]. Third, to address the lack of agreement on trust in AI in health care among experts, our work focused on expert agreement, leaving out some other relevant stakeholders. As the backgrounds of our participants highlight, some stakeholders were actively involved, from developers to practicing clinicians, but others were not included, such as patients, nurses, or hospital administrators. Further work is needed to address this gap.

There were 2 additional limitations more conceptual in nature, and we thank an anonymous reviewer for flagging these. First, our framework does not distinguish between trust *before* and trust *during* use of an AI-based system, yet causal and framing factors of trust may evolve with the experience of using a technology over time. In fact, all our case studies focus more on the initial adoption of a technology and less on trust evolving during its use, reflecting the current early-stage integration of AI systems into health care. That being said, we do hold that our framework can adequately reflect the relevant factors of trust in different AI systems in health care—even if they may change over time. Second, our work did not focus on the phenomenon of distrust despite its undebatable influence on user acceptance and its irreconcilability with trust. We consciously omitted distrust as it is often considered to not be a mere absence of trust but a more complex, richer phenomenon [19,23,95]. Therefore, we believe that a consensus on distrust deserves a paper of its own. However, it should be noted that distrust toward a specific technology, or potentially against AI in general, can of course play a crucial role in inhibiting trust building at a causal level.

Finally, when considering wider conceptual work on trust and the focus on warranted trust in this paper, we need to acknowledge a body of literature that understands trust to be motivated by emotions rather than calculated decisions [128]. The conceptual thinking in this paper leans more toward a cognitive approach to trust building. Thereby, the effect of affective states on trust might be somehow undervalued. However, these affective states can play an important role in trusting and accepting AI in medicine, although their contribution to trusting AI can vary greatly across different instances of human-AI interactions [129]. Therefore, it will be necessary to extend the conceptual thinking beyond cognitive approaches to encompass traits of affective trust.

In summary, our findings suggest that achieving trustworthy AI systems in health care requires a multifaceted approach bridging human-centered and technology-centered approaches. While regulatory precision and boundaries help provide the legal basis needed to develop trustworthy AI, and while technological design features are critical to successful AI development, communication and positive lived experiences with AI systems are at the forefront of user trust. From a user perspective, especially for those who are not AI aficionados, trustworthy technological features and legal compliance should be a sure thing, whereas communication and positive experiences point beyond the technical sphere of AI.

### Conclusions

This paper examines 5 diverse AI systems in health care, revealing the complex landscape of trust in this field. These cases highlight how AI in health care is influenced by various environments, actors, and framing factors. While discussing trust in medical AI in a broad sense may serve as an initial overview, the specific nuances uncovered in these 5 cases demand a more detailed understanding to characterize trusting AI in health care.

Ultimately, however, focus should shift toward ensuring that institutions implement these tools in a *trustworthy* manner rather than merely aiming to indefinitely increase *trust*—only warranted trust is needed for valuable AI implementation. This approach recognizes the complexity of trust dynamics, the risk of superficial increase in trust without a substantial enhancement of trustworthiness, and the importance of trustworthiness in health care institutions. Despite all domain-specific variations, addressing these challenges and embracing transparency and accountability by design can help build a foundation of trust that will underpin the successful integration of AI into health care for the benefit of patients, physicians, and society at large.

## Data Availability

The datasets generated during and/or analyzed during this study are available from the corresponding author on reasonable request. The asynchronous consensus-building process was tracked in repeatedly updated documents.

## Abbreviations

AI: artificial intelligence
BCI: brain-computer interface
ICU: intensive care unit
